# Numerical Modeling of Unreinforced Masonry Walls Strengthened with Fe-Based Shape Memory Alloy Strips

**DOI:** 10.3390/ma14112961

**Published:** 2021-05-30

**Authors:** Moein Rezapour, Mehdi Ghassemieh, Masoud Motavalli, Moslem Shahverdi

**Affiliations:** 1 School of Civil Engineering, University of Tehran, 16th Azar Street, Tehran 11155-4563, Iran; moein.rezapour@alumni.ut.ac.ir (M.R.); m.ghassemieh@ut.ac.ir (M.G.); masoud.motavalli@empa.ch (M.M.); 2 Swiss Federal Laboratories for Materials Science and Technology, Empa, Überlandstrasse 129, 8600 Dübendorf, Switzerland

**Keywords:** unreinforced masonry wall, shape memory alloy, iron-based shape memory alloy, post-tensioning, cyclic loading, retrofitting

## Abstract

This study presents a new way to improve masonry wall behavior. Masonry structures comprise a significant part of the world’s structures. These structures are very vulnerable to earthquakes, and their performances need to be improved. One way to enhance the performances of such types of structures is the use of post-tensioning reinforcements. In the current study, the effects of shape memory alloy as post-tensioning reinforcements on originally unreinforced masonry walls were investigated using finite element simulations in Abaqus. The developed models were validated based on experimental results in the literature. Iron-based shape memory alloy strips were installed on masonry walls by three different configurations, namely in cross or vertical forms. Seven macroscopic masonry walls were modeled in Abaqus software and were subjected to cyclic loading protocol. Parameters such as stiffness, strength, durability, and energy dissipation of these models were then compared. According to the results, the Fe-based strips increased the strength, stiffness, and energy dissipation capacity. So that in the vertical-strip walls, the stiffness increases by 98.1%, and in the cross-strip model’s position, the stiffness increases by 127.9%. In the vertical-strip model, the maximum resistance is equal to 108 kN, while in the end cycle, this number is reduced by almost half and reaches 40 kN, in the cross-strip model, the maximum resistance is equal to 104 kN, and in the final cycle, this number decreases by only 13.5% and reaches 90 kN. The scattering of Fe-based strips plays an important role in energy dissipation. Based on the observed behaviors, the greater the scattering, the higher the energy dissipation. The increase was more visible in the walls with the configuration of the crossed Fe-based strips.

## 1. Introduction

Unreinforced masonry structures comprise a significant part of the world’s existing structures, and some of these structures are even of high cultural and architectural value in their home countries. A considerable portion of the world’s population, especially in developing countries, are inhabitants of such unreinforced masonry structures. Large-scale earthquakes in recent years have caused extensive damage to these structures. Therefore, retrofitting these structures is a global issue that needs to be addressed. The in-plane resistance strength of a masonry structure is much compared to the out-of-plane resistance. In a masonry wall, the out-of-plane resistance mainly depends on the weight of the wall. In this way, the weight of the wall resists the overturning moment applied out-of-plane to the wall. Because the masonry structure’s main strength is due to the in-plane resistance, the research conducted in this field has been mainly focused on in-plane behavior.

The recent progress on the wall systems is performed to replace the traditional concepts. Sharda et al. studied several full-scale modular walls under axial compression to investigate critical design parameters. Their study showed the high potential of fiber materials for the wall systems [1]. Ferdous et al. proposed a new wall system using pultruded glass fiber reinforced polymer composites [2]. Their results demonstrated that the span-to-depth ratio plays an important role in determining failure modes and ultimate capacities of double-H-plank and round-pile specimens. Porto et al. used low-diameter steel reinforcements and reinforced a wall horizontally to improve the masonry walls’ in-plane behavior between the bricklayers [3]. According to Gouveia and Lourenco’s experiments, the use of coils to integrate the wall can increase the lateral strength and ductility of the unreinforced masonry walls by up to 30% [4]. Nateghi and Alami examined the effect of the wall’s dimensions on the type of failure and based on experiments. They showed that the failure mode and crack distribution in the wall depends directly on its dimensions [5]. Pujol et al. studied the effect of masonry materials in filling the space of the frame. For this purpose, a two-story bending frame structure in which its two openings were filled by masonry walls was tested on a seismic table [6]. According to their studies, these walls suffered a sharp drop in strength after structure yielding. Maheri et al. studied two masonry walls, one with vertical mortar and the other without vertical mortar. The results showed that the use of vertical mortar significantly increased the stiffness and in-plane strength of unreinforced walls [7]. To provide the data needed to improve the numerical models of unreinforced masonry walls, Beyer et al. studied the effect of boundary conditions on masonry walls’ behavior [8,9,10,11]. The main failure observed in masonry walls is shear fractures, which are usually diagonal. This failure is very brittle and has little ductility. According to Sadeghi’s research, increasing the axial load on the wall can increase its shear strength and decrease its displacement capacity. He also observed that a high increase in the compressive stress by post-tensioning might change the failure mode to diagonal cracking [12]. This axial load increment can be introduced by post-tensioning elements such as steel bars/tendons in the wall.

There exists vast data about the advantages of post-tensioning in masonry structures. Schultz et al. studied the effect of post-tensioning on the out-of-plane behavior of wind-loaded masonry walls. They observed that by applying post-tensioning, the wall showed suitable strength to wind loading [13]. Kohail et al. examined the effect of post-tensioning on a specific type of masonry shear wall [14]. Soltanzadeh et al. also studied the effect of post-tensioning on the infilled RC frames [15]. Laursen et al. researched the appropriate seismic design criteria for a post-tensioned structure at the University of Auckland [16]. They also proposed a macroscopic model to simulate the cyclic behavior of the post-tensioned masonry wall. Farshchi et al. proposed a general analysis tool to predict the in-plane behavior of the masonry walls. They investigated the effects of post-tensioning on the lateral response of these walls [17,18]. Their results indicated that the axial force improves strength and decreases lateral ductility. They also investigated the effect of brick size and mortar on URM walls’ in-plane behavior [19]. Their studies were limited to the effect of post-tensioning with tendons on in-plane behavior and illustrate an improvement in the behavior of the masonry walls.

Besides the steel material, other materials such as shape memory alloys (SMAs) can be used to post-tension the structures. SMAs are materials that can return to their original shape by heating them to a target temperature after they undergo permanent deformation. By using this feature, these alloys can easily create post-tension stress [20]. Therefore, many studies have been conducted on the post-tensioning of various structures and elements by these materials. In some studies, Shahverdi et al. have examined the possibility of using a form of Fe-SMA that is also economically viable in the post-tensioning of concrete beams [21,22,23,24]. Izadi et al. also examined the possibility of using these alloys’ reversibility capability in metal strips and a metal bridge [25,26,27,28]. Shahverdi et al. evaluated and tested a new type of Fe-SMA reinforcement for post-tensioning several concrete beams [29]. The Fe-SMA reinforcements used in this experiment were ribbed rebar and installed under the embedded concrete beam by the shotcrete concreting process. In a study, Schranz et al. investigated the possibility of using Fe-based corrugated rebar to create post-tension stress [30,31]. Based on the results, these rebars created post-tension stresses of about 350 MPa, which is practically useful for some construction activities.

Making post-tension in the masonry walls is usually performed by vertical elements, and it is difficult to use diagonal elements to implement post-tensioning. Therefore, few studies have been performed on the post-tensioned masonry walls with diagonal elements. Strips made of shape memory alloys make it possible to create a diagonal post-tensioning with an easy practical method. Therefore, in this study, the diagonal and vertical elements with different rates of distribution are used to post-tension the masonry walls. In the current study, to investigate the application of Fe-SMAs to improve the response of masonry walls, finite element models of a masonry wall reinforced with Fe-SMA strips in the different configurations are presented. The numerical models were validated based on existing experimental results. Two types of vertical and cross post-tensioning were applied to the wall reinforced by Fe-SMAs. Cyclic loading was then applied to all models, and their performance was compared with each other.

## 2. Fe-Based Shape Memory Alloy

**Figure 1:**
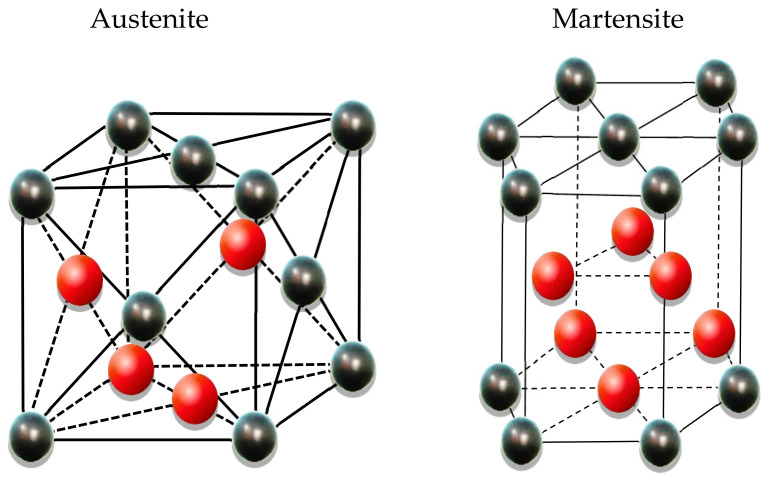
Phase transformation of the Fe-based SMA.

One of the most important features of SMAs is their return to the original shape by applying some heat. Using this feature, the alloy will generate stress to create post-tensioning in a parent structural element. The amount of post-tensioning force depends on the percentage of alloys’ large deformation and heating rate [32]. In general, these materials have two atomic structures, austenite and martensite. These structures can be converted to each other by applied temperature and/or stress (Figure 1) [33]. The shape memory effect of the Fe-SMAs is attributed to the stress-induced martensite transformation from a parent γ-austenite phase to an ε-martensite phase at low temperature. The transformation from the martensite phase to the austenite phase is only possible at high temperatures [34].

**Figure 2:**
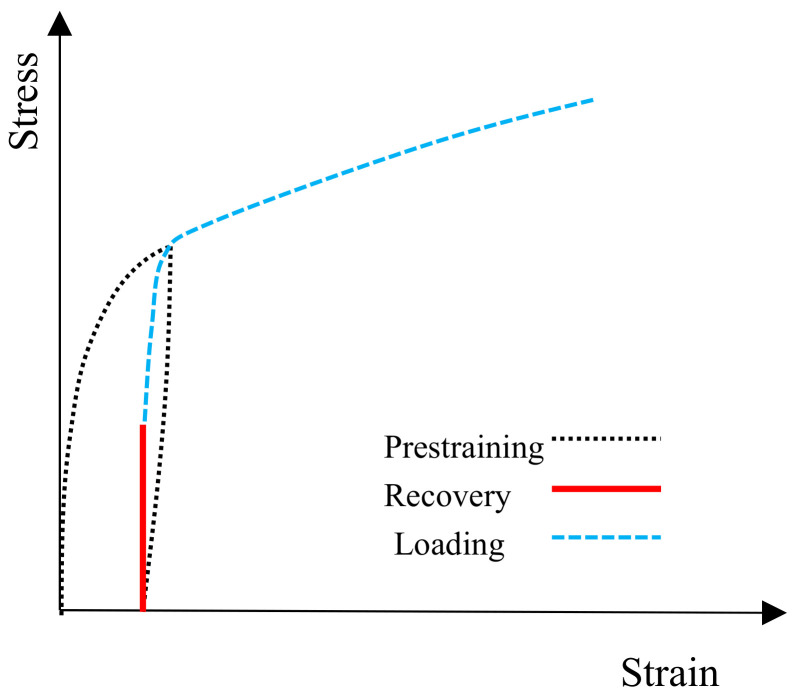
Stress-strain curve of activated Fe-SMA.

When an SMA reinforcement is deformed and heated afterward, its deformation tends to return to zero. This feature can be limited in two ways. The first case is the application of heat by applying constant stress, and the second case is the application of heat with fixed deformation. Figure 2 shows the second case’s behavior diagram, in which deformation is kept constant in the alloy after unloading. By applying heat, the stress in the alloy increases and reaches specific stress. Depending on the degree of fixing deformation and the applied heat, the alloy’s stress varies [20]. In this paper, this stress is used to create post-tension force in the masonry wall.

## 3. Masonry Wall: A Case Study

**Figure 3:**
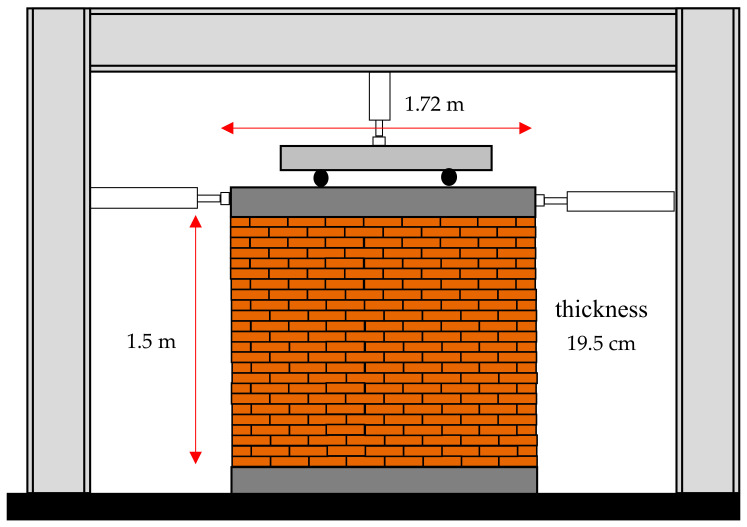
Dimension of experimental masonry wall.

In this study, to investigate the effect of SMA post-tensioning on masonry walls’ behavior, a masonry wall examined experimentally by Karimi et al. is numerically simulated and studied further [35]. The masonry wall studied by Karimi et al. had a 1.5 m height, a 1.72 m length, and a 19.5 cm thickness (Figure 3).

**Figure 4:**
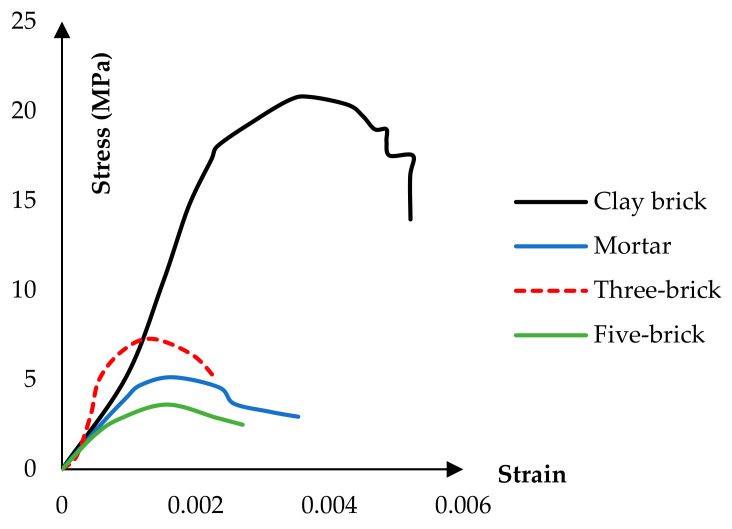
The axial compressive diagram.

The masonry wall was made of clay bricks with dimensions of 45 × 195 × 195 mm, and the thickness of the mortar was about 15 mm. The compressive strength and elastic modulus of clay bricks based on Karimi’s laboratory tests were 20.7 MPa and 8.40 kPa, respectively. The compressive strength and modulus of the mortar’s elasticity between the masonry elements are 5.13 MPa and 3.18 kPa, respectively. Figure 4 shows the test specimens subjected to axial compressive loading, in which examples of single, three, and five bricks are illustrated.

**Figure 5:**
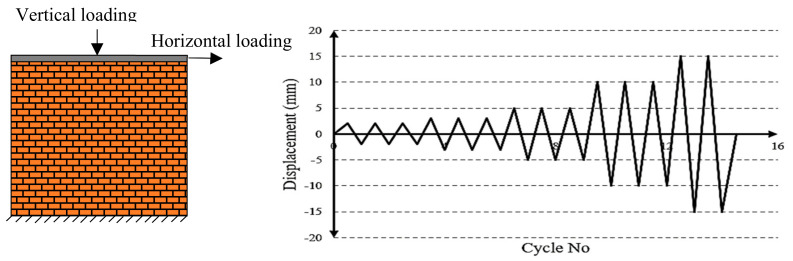
The protocol of the applied lateral displacement, adapted from [35].

The masonry wall in the laboratory was subjected to cyclic loading. For this purpose, a 58 kN vertical load was applied to the wall by a hydraulic jack. This load modeled the weight of the wall above, and it was considered constant during the loading. The horizontal load was applied to the wall, as demonstrated in Figure 5 by load-displacement hybrid control [35]. The amplitude of the major part of the loading was higher than the early part. That is because in cyclic loading protocol, in large deformations, the damage is usually distributed more rapidly in the structure [36,37].

## 4. Numerical Modeling

In the current study, Abaqus finite element software has been used to investigate the effect of pre-stressing SMA on masonry walls’ behavior.

**Figure 6:**
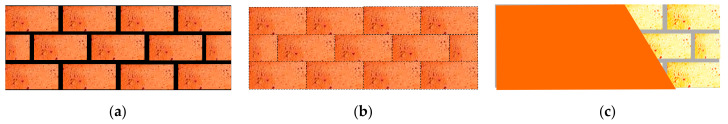
Finite element modeling approaches: (**a**) detailed micro-model, (**b**) simplified micro-model, (**c**) macro-model.

The masonry wall can be modeled either microscopically or macroscopically. In general, there are three modeling methods for masonry walls, two of them are microscopic, and the last one is macroscopic. Figure 6 shows a schematic of these three modeling methods [38]. In the detailed micro-model (Figure 6a), all the details of the masonry units, mortar, and interaction between them must be completely modeled. A simplified micro-modeling approach (Figure 6b) can be a suitable alternative. In this method, the masonry units are expanded by adding the mortar thickness, and the mortar is simulated by the cohesive interaction between the expanded masonry units. This model can simulate how the crack spreads like the previous model, but its accuracy is less than the detailed micro-model. In the third approach, known as the macro-model, the wall’s behavior is considered an integrated, homogeneous, and brittle material. The macroscopic model’s main advantage over microscopic models is that running analyze time is much less than the other models. This method cannot effectively predict the spread of crack, but it is acceptable for studying overall wall behaviors such as static base shear [39]. Since, in this paper, only the cyclic behavior of the wall under static loading is discussed, the macroscopic model has been used to simulate the behavior of the masonry wall.

### 4.1. Material Model

In the current study, the macro-model approach has been used. In this approach, masonry material’s behaviors are simplified so that this material is assumed to be isotropic and homogeneous. The simplified material behavior is similar to that of concrete and exhibits different compressive and tensile strengths. Therefore, the concrete damage plastic (CDP) model has been used for numerical modeling of this isotropic masonry material. This model was introduced by Lubliner and modified by Lee and Fenves [40,41]. CDP is appropriate behavior in Abaqus software to model quasi-brittle materials. The CDP model’s required input includes plasticity, axial compressive behavior, compression damage, axial tensile behavior, and tension damage. In the plasticity part, the dilatancy is controlled by the parameter called dilation angle. Agnihotri et al. proposed that the value of 30° is appropriate for dilation angle in the masonry material and able to generate masonry behavior close to experimental results [42]. Other material properties required for the CDP model were taken as default values (Table 1).

**Figure 7:**
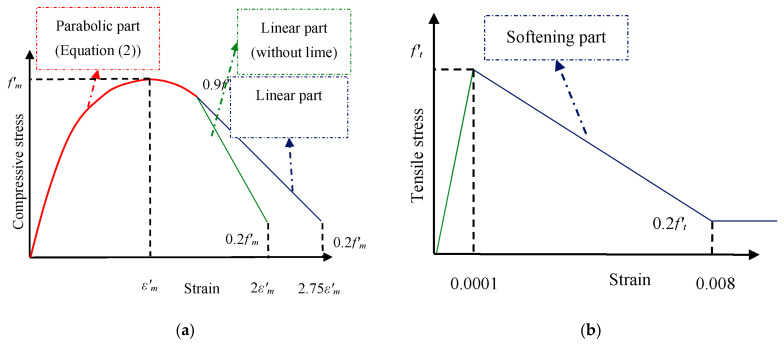
Constitutive law of homogenized masonry (**a**) in compression and (**b**) in tension.

A constitutive model implemented to simulate masonry elements’ compression behavior was proposed by Kaushik et al. [43]. They considered the compressive strength of a masonry prism (consisting of five bricks and four rows of mortar) as a function of brick and mortar’s compressive strength. They presented the following equation to predict the compressive strength of the masonry prism.

(1) $$f_{m}^{\prime }=Kf_{b}{}^{\alpha }f_{j}{}^{\beta }$$

where $f_{b}$ and $f_{j}$ are compressive strength of brick and mortar, respectively. *K*, *α*, and *β* are constants and have been obtained as 0.63, 0.49, and 0.32, respectively [43]. The compressive strength of the masonry prism in Figure 4 is close to the formula presented by Kaushik. Therefore, the constitutive of compressive behavior proposed by Kaushik has been used. This compressive response consists of two parts: the ascending parabolic part and the linear degrading part (Figure 7a). The parabolic part can be expressed in the following non-dimensional equation:

(2) $$\frac{f_{m}}{f_{m}^{\prime }}=2\frac{\varepsilon_{m}}{\varepsilon_{m}^{\prime }}-{\left(\frac{\varepsilon_{m}}{\varepsilon_{m}^{\prime }}\right)}^{2}$$

where $\varepsilon_{m}^{\prime }$ is peak strain corresponding to $f_{m}^{\prime }$. In addition, $\varepsilon_{m}$ and $f_{m}$ are the compressive strain and stress in masonry, respectively. $\varepsilon_{m}^{\prime }$ can be computed from the following equation:

(3) $$\varepsilon_{m}^{\prime }=\frac{0.27f_{m}^{\prime }}{\sqrt[{4}]{f_{j}}{\left(550f_{m}^{\prime }\right)}^{0.7}}$$

According to Figure 7a, Equation (2) was used to depict the ascending stress-strain curve from origin to maximum stress and was extended to the 0.9*f’_m_*. After that, the slope of the linear area depends on the type of mortar. In the macro-model of the masonry wall, a three-line simplified behavior proposed by Agnihotri et al. was used to define the tensile behavior of the homogenized material [42]. In this model, $f_{t}$ is the tensile strength of the mortar and the axial tensile response is shown in Figure 7b. A stress of 0.02 MPa was used in large deformation for convergence in finite element analysis [44].

### 4.2. Damage Parameter

**Figure 8:**
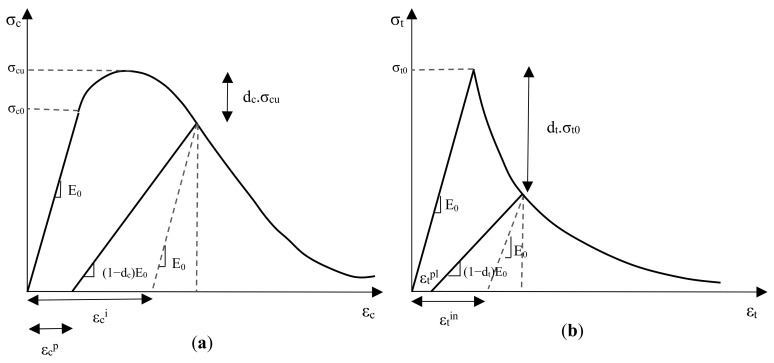
Stress-strain relationship for CDP model (**a**) in compression (**b**) in tension.

In Abaqus software, the CDP model is extensively used to model the damage to concrete structures [45,46]. CDP model can model irreversible damage in brittle materials by reducing stiffness. Stiffness reduction is applied to the material by two parameters, tensile damage (*d_t_*) and compression damage (*d_c_*). Figure 8 shows the axial stress-strain behavior diagram in tension and pressure for concrete materials. In this figure, the model stiffness in loading is reduced by a factor of (1 − *d_c_*) or (1 − *d_t_*) [47,48]. In this form, *ε_t_^in^* and *ε_c_^in^* are the residual tensile and compressive strains in the case of non-damage, respectively, and *ε_t_^pl^* and *ε_c_^pl^* are the residual tensile and compressive strains in the case of concrete damage, respectively. Equations (4) and (5) show the relationships between these strains.

(4) $$\varepsilon_{c}^{pl}=\varepsilon_{c}^{in}-\frac{d_{c}\cdot \sigma_{c}}{(1-d_{c})\cdot E_{0}}$$

(5) $$\varepsilon_{t}^{pl}=\varepsilon_{t}^{ck}-\frac{d_{t}\cdot \sigma_{t}}{(1-d_{t})\cdot E_{0}}$$

where $\sigma_{c}$ and $\sigma_{t}$ are compressive and tensile stress at any point.

### 4.3. Meshing and Boundary Conditions

A three-dimensional cubic element with eight points and reduced integration applied was used (C3D8R) for the masonry wall’s finite element modeling in the current study. The dimensions of these elements are considered to be 50 × 50.6 × 48.75 mm. the number of the elements and nodes are 4080 and 5425, respectively. To apply the boundary conditions, these conditions were investigated experimentally, as shown in Figure 3. The experiment includes a steel frame, specimen, and loading system. The foundation of the wall is connected to the ground through a steel beam. The stiffness of this steel beam can be considered rigid compared to the stiffness of the wall. Therefore, in numerical modeling, the masonry wall foundation is considered rigid. Hence, instead of modeling the foundation, the degree of freedom at the wall bottom is fixed in three directions. Vertical and horizontal loads are applied to the sample by a steel beam drilled on top of the masonry wall. This steel beam’s stiffness is very high compared to the wall’s stiffness, so vertical and horizontal loads can be considered uniformly on the wall’s upper surface. Therefore, instead of modeling steel beams, a rigid body and coupling to the reference point were used to reduce the degree of freedom in the numerical model.

In the experiment, the lateral cyclic loading is applied by two hydraulic jacks on two sides of the specimen. Each complete cycle consists of two half-cycles applied by two left and right hydraulic jacks. The first half-cycle loading begins from left to right by the left jack, and the second one begins from right to left by another jack. In the current study, coupling constraints have been used to model uniform loadings. This coupling constrains the motion of a surface to the motion of one point. The top plate of the wall is coupled to a control point in the middle of the top of the wall, and the lateral cyclic loading is applied to this point. Since the behavior of U-shaped hard profiles above the wall is assumed to be rigid, a 58 kN vertical concentrated load is applied to the top of the numerical model at a uniform compressive stress of 0.2 MPa on top of the numerical model.

### 4.4. FE Models Verification and Mesh Sensitivity Analysis

**Figure 9:**
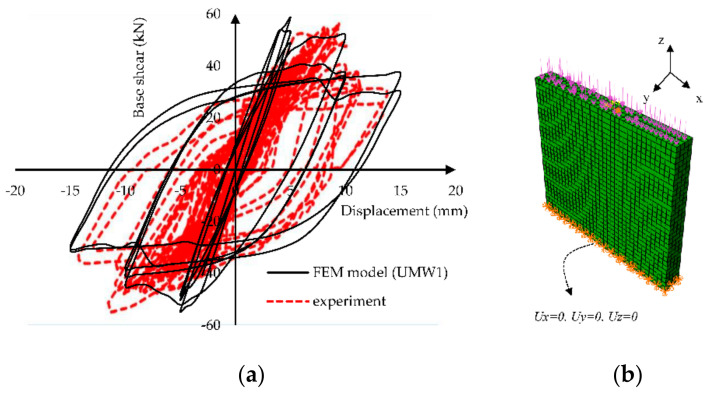
Verification of the finite element macroscopic model results.

**Figure 10:**
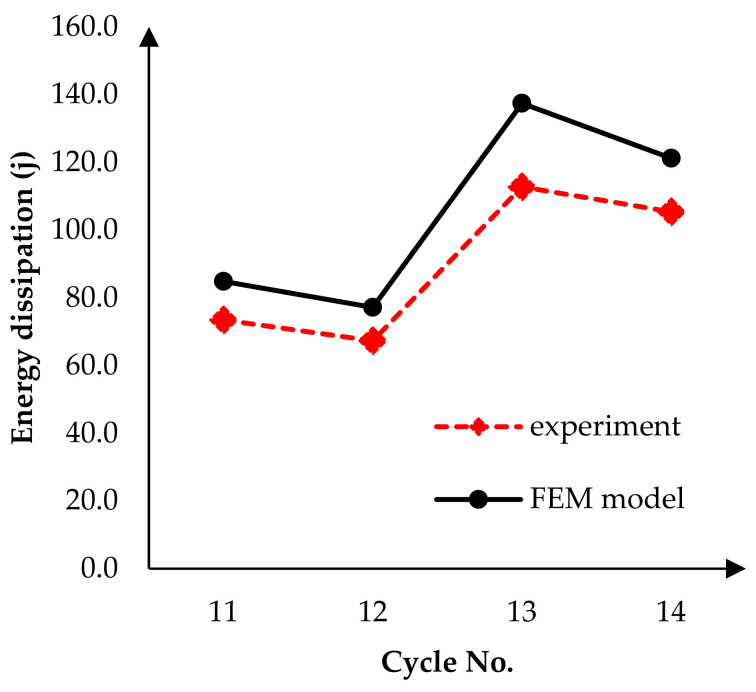
Energy dissipation at the last four cycles.

After modeling the boundary conditions and loading, a nonlinear analysis was performed under cyclic loading. The results are shown in Figure 9, and the experimental model results in terms of the base shear vs. displacement. Figure 10 shows the amount of energy dissipation in the experiment and FEM model at the last four cycles. The difference between these models is about 13% to 17%. According to this graph, the proposed model has a relatively acceptable match to experimental results.

**Figure 11:**
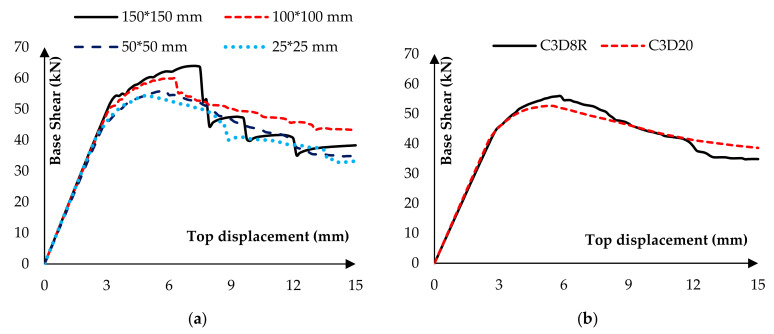
Comparison of different models to validate sensibility (**a**) different sizes (**b**) different types.

To perform mesh sensitivity analysis, the base shear of the wall in multiple meshing conditions was compared. Four mesh sizes of 150, 100, 50, and 25 mm were considered for this comparison. As shown in Figure 11a, decreasing the mesh size converged the wall response. Hence, 50 mm mesh size was chosen because of its low degree of freedom (DOF) and its appropriate accuracy. The element type effect was another parameter that was investigated. The quadratic element response (C3D20) was compared with the 8-node linear element (C3D8R). C3D20 element led to a more precise response and a considerable running time. Figure 11b illustrates that C3D8R has reasonable accuracy for modeling.

## 5. Fe-Based Strips

In this study, Fe-SMA strips were used to investigate the effect of post-tensioning on masonry walls. These strips are made of Fe-SMA with 1.5 mm thickness and a width of 120 mm. The mechanical specifications of these materials are presented in Table 2. This type of Fe-based alloy must be heated to 160 °C to create post-tension stress. Because these materials are highly electrically resistant, the alloy’s temperature can be easily increased as needed by the electric current.

According to Table 2, this alloy can produce up to about 300 Mpa, ideally in a post-tensioning situation [49]. Due to factors such as creep in concrete, shrinkage, human error in construction, etc., it is assumed that not all the Fe-SMA strips’ potential can be used to create stress. Therefore, in this study, post-tensioning stress is considered to be about 200 Mpa.

**Figure 12:**
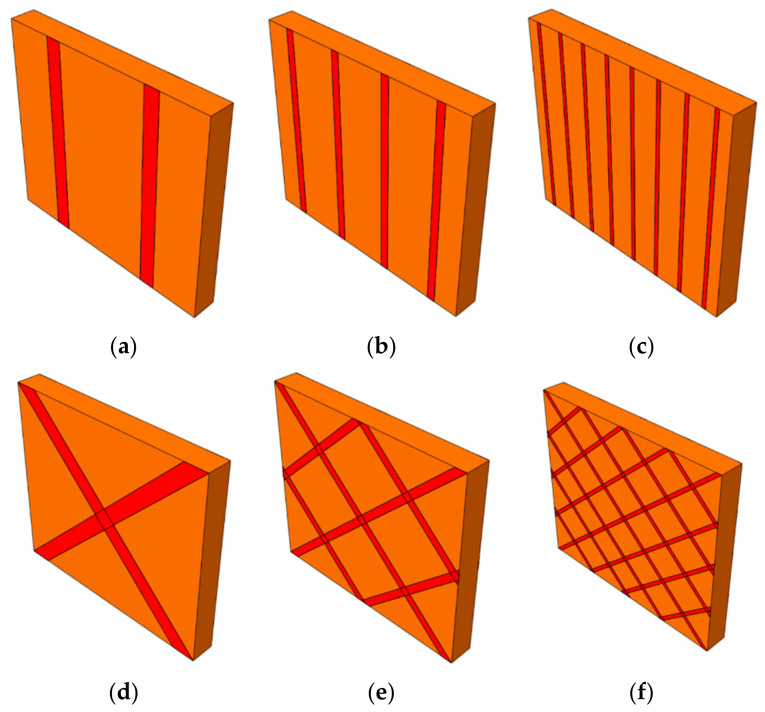
Numerically studied walls, (**a**) UMW2, (**b**) UMW3, (**c**) UMW4, (**d**) UMW5, (**e**) UMW6, and (**f**) UMW7.

In this research, two general methods have been considered for installing the strips on the masonry walls. In the first method, the Fe-SMA strips are installed vertically, and in the second method, they are installed to the masonry wall as a cross. The connection between masonry and strips is bonded by mortar. Therefore, in this study, seven walls with the letters UMW1, UMW2…, and UMW7 are studied. The UMW1 wall is the reference wall on which no strip is applied. According to Figure 12, the UMW2 to UMW4 have vertical stripes, and the UMW5 to UMW7 walls have cross-strips. Table 3 also presents the geometrical properties of the studied models.

## 6. Creating Post-Tension in Numerical Models by Fe-SMA Strips

**Figure 13:**
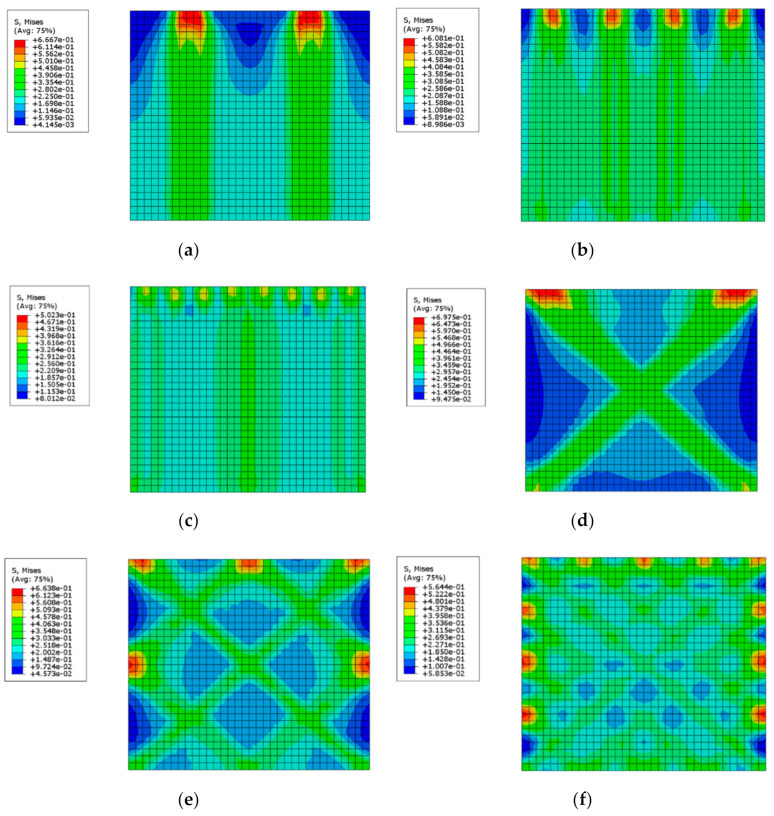
Stress distribution in numerically studied models, (**a**) UMW2, (**b**) UMW3, (**c**) UMW4, (**d**) UMW5, (**e**) UMW6, and (**f**) UMW7.

**Figure 14:**
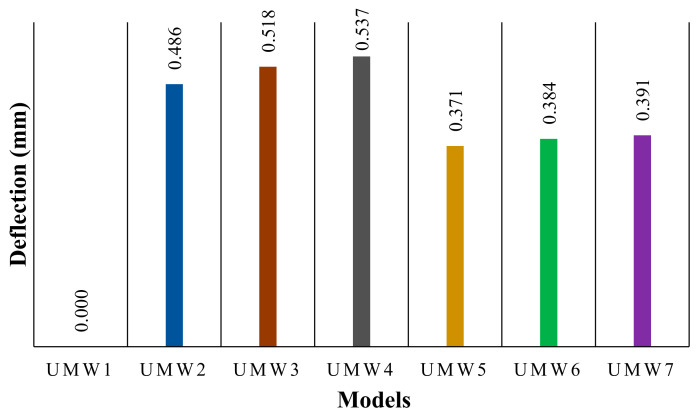
Average deflection of the top of the models.

To embed the SMA strips on the outer surface of the masonry walls, bonded condition tie interaction was used. This interaction completely attached the strip’s surface to the wall and can transfer deformation and stress from the strip to the wall. There are two ways to apply post-tensioning to the SMA strips. In the first method, the coefficient of thermal expansion is assigned to the SMA, and a high initial temperature is applied to the strips. Before starting the cyclic loading, the alloy temperature decreases linearly. Due to this decrease in temperature, the alloy tends to shrink. The tie interaction between the strips and the wall prevents this reduction in deformation, which ultimately causes tensile stress in the strips. In the second method, the stress is applied directly to the strips through predefined fields [50,51]. After assigning the pre-stressing force, an initial step without any loading was defined to establish self-equilibrium. Since the second method is less complex than the first, the second method has been used in this current study. Figure 13 shows the stresses caused by the post-tensioning of SMA in the masonry walls, in which this stress can be transferred to the wall. Tie contact between SMA and wall causes this stress distribution [50]. Accordingly, in models where the strips are more distributed (UMW4 and UMW7), the more effective stress distribution is observed. Figure 14 also shows the average deflection at the top of the wall. According to this figure, the vertical-strip walls (UMW2, UMW2, and UMW3) experienced more deflection than the cross-strip walls. In the vertical-strip walls, the higher the scattering of the strips (UMW4), the higher the deflection is observed. In the cross-strip models (UMW5, UMW6, and UMW7), the amount of deflection is almost equal to each other and less than the vertical-strip walls. Because in the models where the strips are more scattered, the stress is transferred to the wall more effectively, and this has caused more deflection in these walls. In cross-strip walls, due to the diagonal of the strip, less vertical stress is transferred to the wall than vertical strips. Therefore, there is less deflection in the cross-strip models. In numerical modeling, shape memory strips are modeled with shell elements (S4R) and attached to the masonry wall with a tie interaction. A surface-based tie constraint was used to make the translational and rotational motion, as well as all other active degrees of freedom, equal for a pair of surfaces [52].

## 7. Hysteresis Results

**Figure 15:**
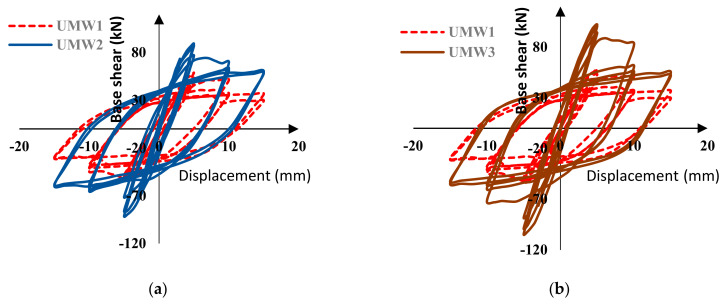

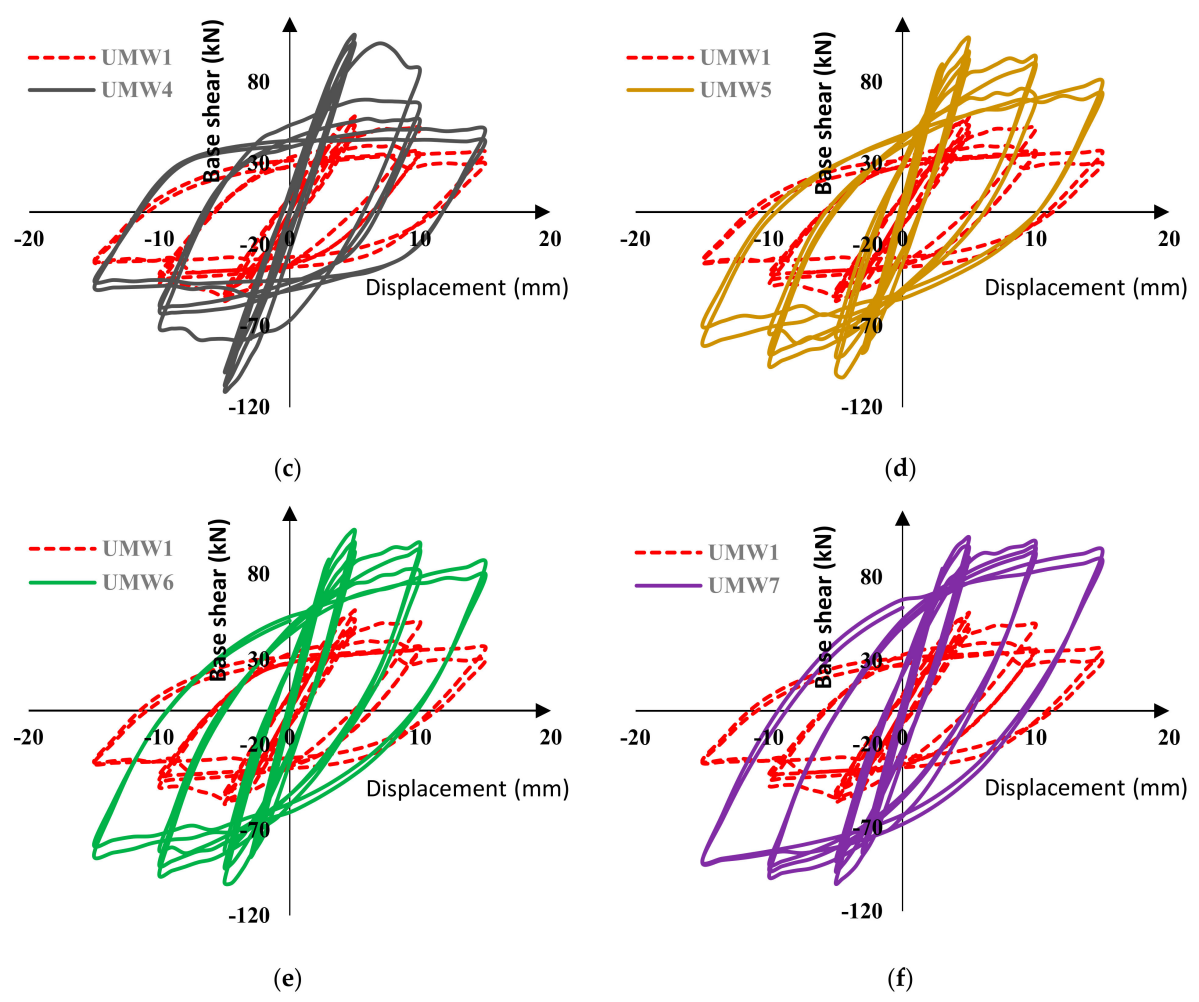
Hysteresis of Fe-SMA reinforced walls, (**a**) UMW2, (**b**) UMW3, (**c**) UMW4, (**d**) UMW5, (**e**) UMW6, and (**f**) UMW7.

To investigate the effect of post-tensioning by Fe-based alloys, all numerical models were subjected to static cyclic loading. Figure 15 shows the cyclic behavior of reinforced walls compared to the unreinforced wall (UMW1). According to these diagrams, the use of Fe-SMA strips has several important advantages, the most important of which is increasing the masonry wall’s strength. As shown, the SMA strips have significantly increased the wall’s strength, increasing the walls’ energy absorption capacity. As the energy absorption property of the masonry walls increases, so does their damping capabilities.

**Figure 16:**
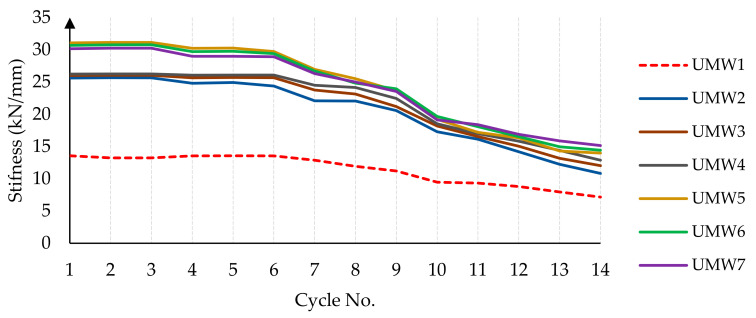
The stiffness reduction in masonry walls during the loading.

As shown in Figure 15, the stiffness of the masonry walls decreases gradually during the loading cycles. This reduction in the stiffness is due to damage taking place in the masonry wall during the loading. Damage applied in the form of tensile (cracking) and compressive (crushing) to the model reduces the stiffness of the material according to the CDP model. In Figure 16, the stiffness of the masonry walls is presented at each cycle. The use of an SMA strip has increased the wall’s stiffness almost twice as much as without strips. In models where SMA strips are embedded crosswise (UMW5, UMW6, and UMW7), the stiffness increases more than in models where SMA strips are embedded vertically (UMW2, UMW3, and UMW4). This is because diagonal strips contribute more to the lateral stiffness of the wall than vertical strips. The increase in stiffness in the UMW5, UMW6, and UMW7 models is since, in these models, SMA strips play a more effective role in creating lateral stiffness than the strips in the UMW2, UMW3, and UMW4 models.

**Figure 17:**
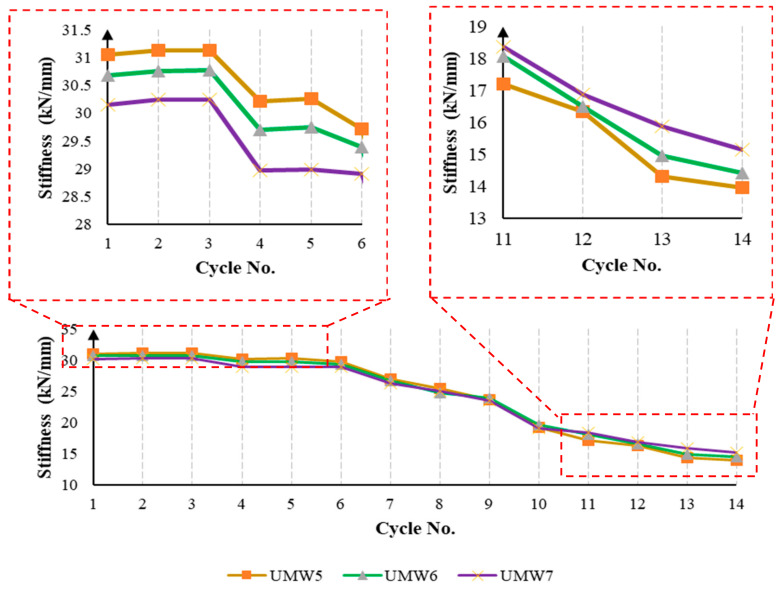
The stiffness reduction in the cross-strip masonry walls during the loading.

In cross-strip models, the stiffness of masonry walls is almost similar to each other, and it can be concluded that the scattering of SMA strips throughout the wall has a minor effect on its stiffness. However, if in Figure 17 the range of vertical and horizontal axis numbers is more limited, in the initial loading cycles, the model in which the SMA strips are less distributed (UMW5) has more stiffness. However, in the final cycles, the trend is reversed, and the model in which the SMA bar is more distributed (UMW7) has more stiffness than other models. This phenomenon is that the scattering of the SMA strip reduces the amount of damage in different places, and this causes the stiffness level in UMW7 to be higher than others in large deformations. However, in small deformations in UMW5, due to wider cross-strips in the four corners of the wall, more stiffness is observed than in other models.

**Figure 18:**
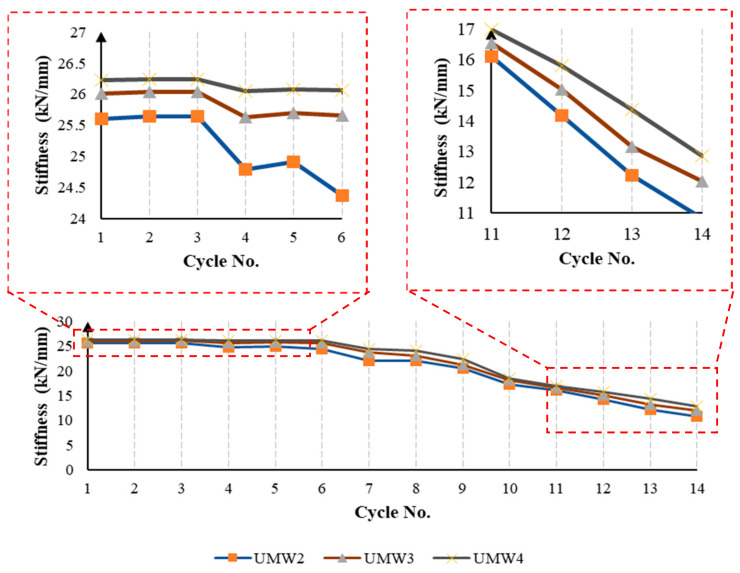
The stiffness reduction in the vertical-strip masonry walls during the loading.

As shown in Figure 18, in the vertical-strip models, the models’ stiffness is close to each other. However, if in Figure 18 the range of vertical and horizontal axis numbers is more limited, in the initial loading cycles, the stiffness of the model in which the SMA strips are more distributed (UMW4) is higher than other models. In the final cycles, this model’s stiffness (UMW4) is still greater than other models. However, the stiffness difference between this model and others is raised. This phenomenon is that the scattering of SMA strips in the model reduces the amount of damage in different places, and this causes that the amount of stiffness in the UMW4 wall is more than in other models in large deformations.

**Figure 19:**
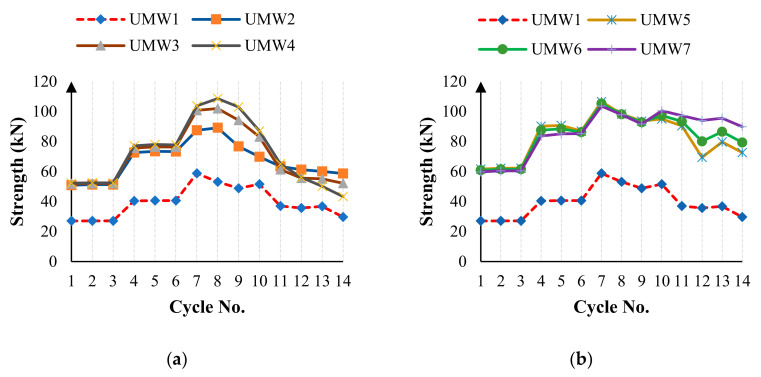
Maximum strength in each cycle of the loading in the positive direction (**a**) vertical-strip walls and (**b**) cross-strip walls.

As shown in Figure 15, the wall strength decreases during the loading. The rate of this decrease varies in different models. Normally, in all models, the maximum strength occurs in the seventh cycle, after which the strength begins to decrease. Figure 19 shows the maximum strength in each cycle of loading in the positive direction for different models. According to Figure 19a, cross-strip models have the highest strength to lateral load. In the vertical-strip models, the UMW4 model has the highest strength in the seventh cycle than the other vertical-strip models. However, in the final cycles, the strength of the UMW4 model drops more than others, and in total, the UMW2 model has the highest strength in the fourteenth cycle. According to this figure, in the vertical-strip models, the higher the scattering of the strips, the higher the yielding strength, but the lower the ultimate strength. In cross-strip models, the strength in the seventh cycle of loading is approximately equal to each other, and the scattering of the strip has a relatively small effect on the strength in the seventh cycle. However, if the precision level is raised, it will be seen that the UMW5 model has more strength in the seventh loading cycle than others. In larger deformations, the strength of the UMW5 model decreases significantly compared to other models. According to Figure 19b in cycles 13 and 14, the strength of the UMW7 model is higher than other models. Therefore, in cross-strip models, the strips’ scattering rate has a negligible effect on the yield strength, but in large deformations, the higher the scattering of the strips, the higher is the ultimate strength.

**Figure 20:**
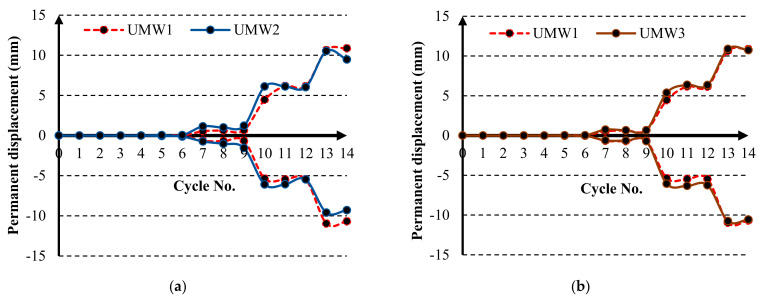

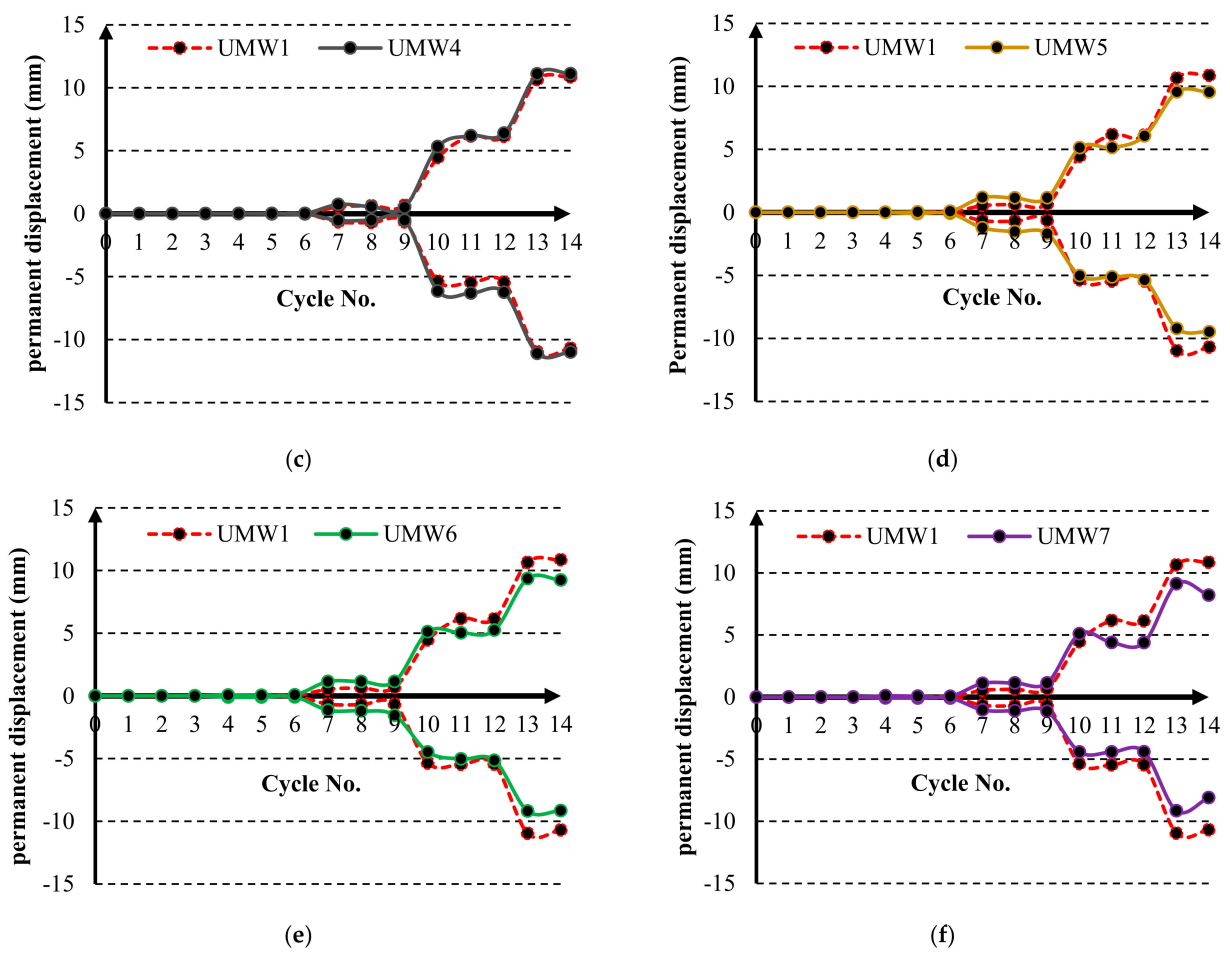
Permanent deformation during the cyclic loading for models, (**a**) UMW2, (**b**) UMW3, (**c**) UMW4, (**d**) UMW5, (**e**) UMW6, and (**f**) UMW7.

Permanent deformation in the structure plays an important role in energy dissipation. Figure 20 shows the permanent deformation during the cyclic loading for different models. Positive and negative numbers indicate a permanent deformation in the cyclic loading’s right and left direction, respectively. According to the diagram, most models have a permanent deformation close to the UMW1 model. In vertical-strip walls, the ending cycles’ rate of permanent deformation is close to the UMW1 model. In these models, the lateral strength has not increased enough to reduce the permanent deformation. However, in cross-strip models, the situation is different. In this form, the lateral strength in large deformations has increased dramatically. Therefore, the number of permanent deformations has been reduced to some extent during the loading. Table 4 shows the reduction in the permanent deformation in the different loading cycles for the cross-strip models. According to this table, in cycles 7 to 10, the rate of permanent deformation of SMA-equipped models is higher than in the UMW1 model, and therefore the reduction percentage in Table 4 is considered negative for cycles 7 to 10. According to this table, in cycles 11 to 14 on the UMW7 wall, the rate of permanent deformation has shown the greatest decrease. Therefore, cross-strip walls have less permanent deformation than vertical-strip walls, and the higher the scattering of SMA strips, the lower the permanent deformation rate.

**Figure 21:**
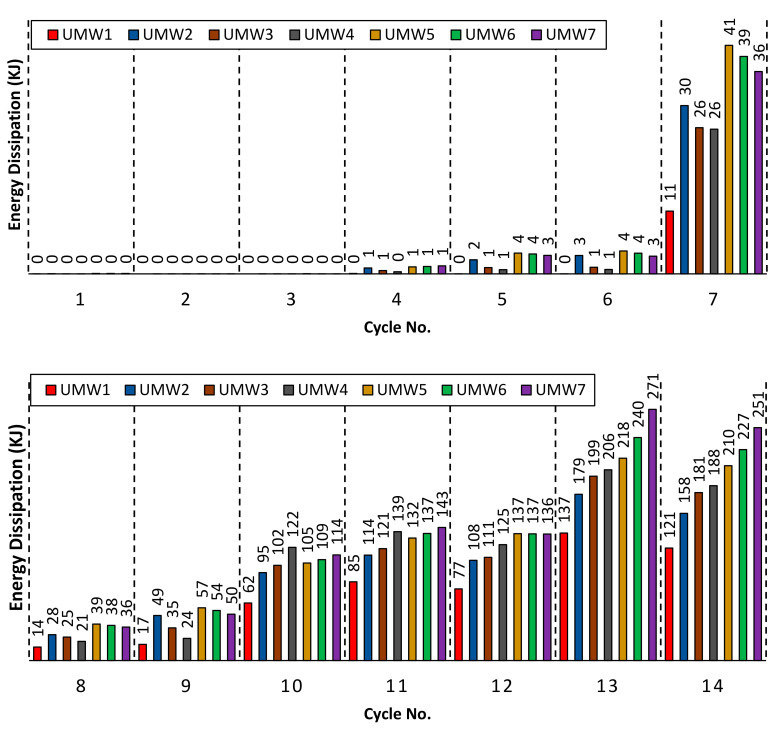
Energy dissipation at each load cycle.

The amount of energy dissipation is directly related to the amount of lateral strength and permanent deformation of the structure. Figure 21 shows the amount of energy lost during the cyclic loading. Since the masonry wall’s behavior is almost linear in the early cycles, the energy dissipation rates up to six cycles are close to zero. By creating a plastic deformation in the system, the amount of energy dissipation increases. Walls reinforced with Fe-SMA strips have been able to increase the energy dissipation of the wall. As shown in Figure 21, the cross-section walls have shown more energy dissipation than other models. In cross-strip models, the resistance, especially in the last cycles, is higher than other models, which increases the energy dissipation capacity.

**Figure 22:**
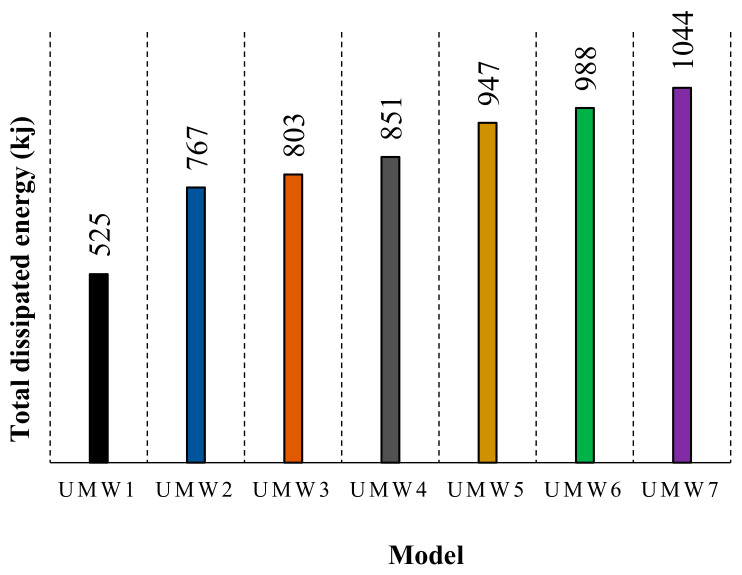
Total energy dissipation of each model.

Figure 22 shows the total energy dissipation for all models. In this figure, each of the graph columns is equal to the sum of the energies dissipated in all the hysteresis cycles for a numerical model. According to this figure, the UMW7 model has the highest energy dissipation. In the vertical-strip models, the UMW4 model has the highest energy dissipation. Therefore, the higher the scattering of SMA strips on the masonry wall, the higher the energy dissipation.

## 8. Conclusions

In this study, the effect of post-tensioning masonry walls by Fe-SMA strips was investigated. The Fe-based alloys were installed in the form of crosses and vertical strips in walls, and post-tension stress was applied to them. Based on the analysis, the results can be described as follows.

In vertical-strip walls, the effect of post-tensioning in creating stress in the masonry wall is more than the cross-strip walls. The vertical-strip SMAs could apply more vertical pressure to the wall, and the higher the scattering of the vertical strips, the more efficiently the stress is distributed in the wall. In cross-strip walls, the scattering of the strips has a negligible effect on the deflection of the wall, but it makes the stress distribution process in the wall more uniform.

The use of Fe-based strips increases the stiffness of the specimens relative to the reference wall. Cross-strip models have more stiffness than vertical-strip models. So that in the vertical-strip walls, the stiffness increases by 98.1%, and in the cross-strip model’s position, the stiffness increases by 127.9%. The dispersion of Fe-based strips has little effect on lateral stiffness. Of course, if the precision is increased, the more distributed the strips on the cross-strip walls, the stiffness in small and large deflections are reduced and increased, respectively. However, in the vertical-strip models, the strips’ scattering increases the stiffness of the wall in both small and large deformations.

The use of Fe-based strips increases the strength of the masonry wall. In the vertical-strip walls, the yield strength in the model in which the scattering of the Fe-based strip is higher increases the level of the yield strength, but in larger cycles, its strength decreases sharply. In cross-strip models, the lateral strength is greater than the vertical-strip models, and the scattering of the strips has little effect on the yield strength. In the vertical-strip model, the maximum resistance is equal to 108 kN, while in the end cycle, this number is reduced by almost half and reaches 40 kN. In large deformations, the greater the scattering of the Fe-based strips, the more resistant the masonry wall is. So that in UMW7, the maximum resistance is equal to 104 kN, and in the final cycle, this number decreases by only 13.5% and reaches 90 kN.

The use of strained strips has little effect on the permanent deformation. In cross-strip models in large load cycles, the permanent deformation rate is somewhat lower than the reference wall.

One of the most important parameters of a structure is its energy dissipation. Based on the results obtained, the use of these strips has greatly increased the energy dissipation of the structure and has been able to double the energy dissipation in some models. In cross-strip models, the energy dissipation is higher than in vertical-strip models. UMW2 absorbed energy 46% more than UMW1, and this number is about 80.4% in UMW5. Another important parameter that plays an important role in energy dissipation is the scattering of Fe-based strips. Based on the observed behaviors, the greater the scattering, the higher the energy dissipation becomes.

## Figures and Tables

**Table 1:** Plastic properties of the homogenized masonry.

Dilation angle	30°
Flow potential eccentricity	0.1
The ratio of initial equibiaxial compressive yield stress to initial uniaxial compressive yield stress	1.16
The ratio of second stress invariant	0.67
Viscosity parameter	0.001

**Table 2:** Mechanical properties of iron-based memory alloy [49].

Ultimate tensile strength (MPa)	1000
Post-tension stress by heating the element to 160° C (Mpa)	300–400
Pre-strain (%)	4
Elasticity module after activation (Mpa)	70,000

**Table 3:** Geometric properties of numerically studied models.

Models	Length (cm)	Height (cm)	Thickness (cm)	SMA Strip Thickness (mm)	Strip Width (mm)	Center to Center Distance of the Two Parallel Strips (cm)
UMW1	172	150	19.5	-	-	…
UMW2	172	150	19.5	1.5	120	86
UMW3	172	150	19.5	1.5	60	43
UMW4	172	150	19.5	1.5	30	34.5
UMW5	172	150	19.5	1.5	120	…
UMW6	172	150	19.5	1.5	60	57
UMW7	172	150	19.5	1.5	30	28.5

**Table 4:** Permanent deformation of the cross-strip walls.

	Cycle NO	UMW1	UMW5		UMW6		UMW7	
		Permanent Deformation (mm)	Permanent Deformation (mm)	Percentage Reduction	Permanent Deformation (mm)	Percentage Reduction	Permanent Deformation (mm)	Percentage Reduction
Positive deformations	7	0.54	1.18	−119.28	1.15	−113.47	1.10	−105.52
	8	0.63	1.14	−81.00	1.14	−80.65	1.14	−80.17
	9	0.67	1.16	−73.18	1.15	−72.33	1.14	−71.05
	10	4.46	5.13	−15.14	5.12	−14.83	5.10	−14.49
	11	6.17	5.14	16.65	5.03	18.44	4.40	28.71
	12	6.14	6.06	1.23	5.24	14.63	4.39	28.54
	13	10.65	9.56	10.26	9.35	12.16	9.12	14.33
	14	10.86	9.54	12.13	9.24	14.88	8.21	24.37
Negative deformations	7	−0.65	−1.22	−86.32	−1.14	−74.67	−1.03	−57.97
	8	−0.70	−1.53	−118.65	−1.20	−72.00	−1.10	−57.82
	9	−0.66	−1.69	−156.03	−1.55	−135.70	−1.14	−73.74
	10	−5.36	−5.02	6.36	−4.47	16.54	−4.42	17.51
	11	−5.46	−5.12	6.25	−5.03	7.88	−4.42	19.06
	12	−5.45	−5.37	1.55	−5.14	5.60	−4.40	19.20
	13	−10.96	−9.21	15.98	−9.18	16.23	−9.16	16.48
	14	−10.69	−9.48	11.35	−9.16	14.37	−8.10	24.26

## Data Availability

Not applicable.
